# Validation of the translated version of the EVAN-G scale in a Chinese-speaking population

**DOI:** 10.1186/s12871-022-01909-w

**Published:** 2022-11-24

**Authors:** Xinting Wang, Wenjun Lin, Linwei Liu, Zhenyuan Wu, Yushan Wu, Yusheng Yao

**Affiliations:** 1 Department of Nephrology, Fuzhou Hospital of Traditional Chinese Medicine Affiliated to Fujian University of Traditional Chinese Medicine, Fuzhou, China; 2 Department of Anesthesiology, Shengli Clinical Medical College of Fujian Medical University, No.134, Dongjie, Fuzhou, 350001 China

**Keywords:** Patient-reported measures, Patient satisfaction, Postoperative, Scale, Anesthesia

## Abstract

**Background:**

This study aimed to translate the French version of a perioperative satisfaction questionnaire (EVAN-G) scale, a validated questionnaire for assessing perioperative patient satisfaction, into a Chinese version and validate it in Chinese-speaking patients.

**Methods:**

We developed the Chinese version of the EVAN-G (EVAN-GC) scale based on the original French version of the EVAN-G. The EVAN-GC scale, the Short version of the Spielberger State-Trait Anxiety Inventory (S-STAI), and the McGill pain questionnaire (MGPQ) were administered on the WeChat mini program. We invited patients to complete these questionnaires within 4 to 24 h after surgery. The psychometric validation of the EVAN-GC scale included validity, reliability, and acceptability.

**Results:**

Among 220 patients, 217 (98.6%) completed the EVAN-GC scale after surgery. The item-internal consistency revealed good construct validity. Compared with the total scores of the S-STAI and MGPQ, the EVAN-GC scale showed excellent convergent validity (*ρ* = − 0.32, *P* < 0.001; *ρ* = − 0.29, *P* < 0.001). The EVAN-GC scale could differentiate between groups, which showed good discriminate validity. The Cronbach’s alpha coefficient (0.85) of the translated scale demonstrated satisfactory internal consistency reliability, and a 36-patient subsample retest evidenced good test-retest reliability (*ρ* = 0.82, *P* < 0.001). In addition, the median [interquartile range] time of completing the EVAN-GC scale was 3.7 [2.9–4.9] min.

**Conclusions:**

The EVAN-GC scale has good psychometric properties similar to those of the original French version. The EVAN-GC scale is a valid and reliable measurement to assess patient satisfaction in Chinese-speaking patients.

**Trial registration:**

The Chinese Clinical Trial Registry, ChiCTR2100049555.

## Background

Patient satisfaction is always considered a significant influencing factor in health care [1]. Patients with high satisfaction are more likely to follow medical staff instructions, accept treatment and follow-up, contribute to high-quality nursing, reduce adverse events, and improve clinical outcomes [2]. Nevertheless, satisfaction is not merely a simple concept suitable for every patient. It depends on whether the feelings and experience of patients matched their expectations during treatment [3, 4]. Hence, a satisfaction degree is considered an adequate measure of health care, and most hospitals worldwide use it to improve health care quality [5]. The perioperative satisfaction scale measures patients’ satisfaction status in the perioperative period, including the preoperative period, operation period, and postoperative period.

Several perioperative period quality scales or questionnaires have been developed in various clinical specialties and settings, such as the Leiden Perioperative Care Patient Satisfaction (LPPS), the Iowa Satisfaction with Anesthesia Scale (ISAS), and the EVAN-G scale [6–8]. The 26-question EVAN-G scale includes six dimensions (attention, privacy, information, pain, discomfort, and waiting), which are rigorously developed and validated. The EVAN-G scale has been widely used in different studies due to its strong psychometric characteristics and reliability in general anesthesia patients [9–11]. In addition, Barnett’s systematic review of patient satisfaction measures noted a scarcity of well-developed satisfaction questionnaires and highlighted the robustness of the EVAN-G scale, which has been validated in French and Spanish [12, 13].

Furthermore, all the included studies relied on paper-based assessments during the perioperative period. Due to the popularity of WeChat in China, other than the trouble of downloading a new application, scanning the Quick Response Code before completing the web scale is more accessible, acceptable, and convenient. The mini-programs are planted on mobile applications such as WeChat, and our goal is to make a Chinese web version scale on a WeChat-based program [14].

However, in China, perioperative satisfaction scales are rarely developed, translated, and validated. This study aimed to develop and validate a Chinese version of the EVAN-G scale in a Chinese-speaking perioperative patient cohort. We hypothesize that the EVAN-GC scale would have similar psychometric properties and transitional ability in assessing perioperative satisfaction as the original French scale in Chinese-speaking populations.

## Methods

This prospective validation trial was approved by the Ethics Committee of Fujian Provincial Hospital, and written consent was obtained from all participants. The study protocol was registered at the Chinese Clinical Trials Registry (http://www.chictr.org.cn, ChiCTR2100049555, 2/8/2021). We conducted this trial at Fujian Provincial Hospital, Fuzhou, China, in accordance with Consolidated Standards of Reporting Trials (CONSORT) guidelines.

The inclusion criteria were patients aged equal to or over 18 years, scheduled for elective surgery under general anesthesia, and familiar with smartphone use. Patients with cognitive disorders, inability to understand and read Mandarin, known alcohol or substance abuse, completion of a similar questionnaire before, or any conditions that impeded the completion of the questionnaire within 24 h after surgery were excluded.

### Translation of the EVAN-GC scale

The EVAN-G scale is intended to measure satisfactory conditions for perioperative patients. We developed the EVAN-GC scale according to the methods adopted by the International Quality of Life Assessment (IQOLA) project [15]. The translation process from French to Mandarin was forward and backward translation. Above all, the original French EVAN-G scale was translated into Chinese by a Chinese medical student proficient in French and a native Chinese speaker without a medical background. Second, the two translators and a recording observer discussed, modified, and synthesized the first translation graft. Then, another two bilingual French speakers back translated the Chinese version into French. After that, an expert committee discussed discrepancies and produced the prefinal version. Finally, pilot testing on 36 patients was performed to ensure understanding of all questions and ease of administration. No specific cultural adaptation was made after the pilot testing.

### Procedures

An electronic medical record system was used to screen for potential participants. The study procedure and information about the planned surgery were informed orally on the day before surgery. In addition, basic information about the enrolled patients was recorded, such as age, sex, the extent of surgery, and educational background. We classified the extent of surgery as grade I − IV based on a deterministic patient classification approach, which groups patients according to the International Classification of Diseases, 9th Revision, Clinical Modification (ICD-9-CM3) codes [16]. The educational background was classified as follows: junior high school or below, high school, associate’s or bachelor’s degree, and master’s degree or above [17].

On the day of surgery, patients were invited to complete the EVAN-GC scale, the Spielberger State-Trait Anxiety Inventory (S-STAI) [18], and the McGill pain questionnaire (MGPQ) [19] within 4 to 24 h after surgery. The S-STAI is a reliable measurement for clinical screening and behavioral research. The MGPQ contributes to evaluating pain intensity. These scales were performed under the support of the WeChat mini program. The enrolled patients were provided with a link to the questionnaires, and the assessor collected data from the background program. The duration of completing each dimension was recorded. The 26-item EVAN-GC scale was evaluated using a five-point Likert scale, in which 1 point equaled much less than expected, 2 points meant less than anticipated, 3 points stood for as expected, 4 points equaled more than expected, and 5 points equaled much more than expected [20]. In other words, a higher score indicates excellent satisfaction, while a lower score reveals poor satisfaction.

### Statistical analysis

The sample size was calculated based on our 65-patient pilot test. According to the formula N = Z^2^pq/δ^2^ (α = 0.05, δ = 0.07, *p* = 56%, q = 1-p), [21, 22] 193 patients were required for a statistical power of 90% power at the 0.05 significance level. Assuming a 15% dropout rate, 227 patients were deemed for this study.

IBM SPSS for Windows version 25.0 software (SPSS Inc., Chicago, IL, USA) was used for statistical analyses. Data were reported as mean (standard deviation, SD), median [interquartile range, IQR], or number (percentage, %) as appropriate. For Gaussian data, correlations were calculated with the Pearson correlation coefficient, and for non-Gaussian data, correlations were calculated with the Spearman correlation coefficient. Inferential analysis was performed using the Mann–Whitney or Kruskal–Wallis tests.

First, we measured construct validity, convergent validity, and discriminate validity. To validate the construct validity, we identified interitem, item-dimension, interdimensional correlations, and principal component factors. We analyzed them by several methodologies, including Spearman’s correlation coefficient (ρ), principal component analysis, and factor analysis, as appropriate. To calculate the convergent validity, we measured the correlation between the EVAN-G scale and S-STAI and the correlation between the EVAN-GC scale and MGPQ using ρ. To assess the discriminant validity, we calculated the differences between different patient groups (such as age, sex, and educational background) using the Mann–Whitney or Kruskal–Wallis tests. Second, we assessed internal consistency reliability and test-retest reliability. Cronbach’s alpha coefficients were computed to analyze the internal consistency reliability. The test-retest reliability was evaluated by Spearman’s correlation coefficient on a 36-patient-scale subsample estimated twice between a 15-day interval. Third, acceptability and feasibility were valued with successful completion rate and duration for patients to complete the questionnaire.

## Results

**Fig. 1:**
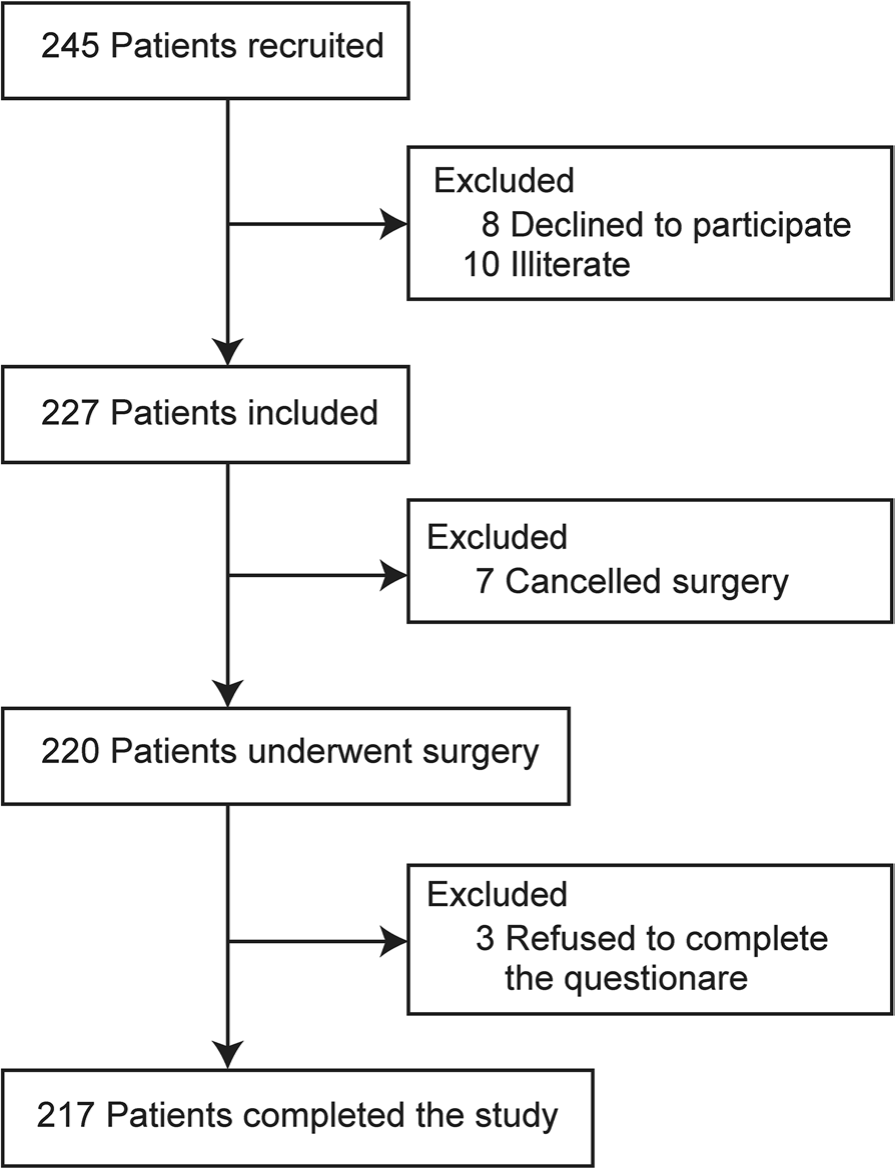
Flow diagram of the study

From August 2021 to October 2021, we screened 245 patients scheduled for elective surgery under general anesthesia. Eight patients declined to participate, and ten were excluded due to being illiterate. After recruitment, seven patients were excluded before postoperative follow-up, and three refused to complete the EVAN-GC questionnaire after surgery (Fig. 1). The median (IQR) patient age was 49 [41–57]. Surgical procedures were classified as grade I (3.2%), grade II (41.9%), grade III (33.2%), and grade IV (21.7%). Other baseline variables are presented in Table 1.

**Table 1:** Patient and perioperative characteristics (*n* = 217)

Variable	Value
Age, years	49 [41–57]
Male, n (%)	87 (40.1%)
Extent of surgery, n (%)	
Grade I	7 (3.2%)
Grade II	91 (41.9%)
Grade III	72 (33.2%)
Grade IV	47 (21.7%)
Educational background, n (%)	
Junior high school or below	69 (31.8%)
High school	96 (44.2%)
Associate degree or Bachelor’s degree	47 (21.7%)
Master’s degree or above	5 (2.3%)
Previous anesthesia, n (%)	
Yes	127 (58.5%)
No	90 (41.5%)

Data are presented as median [interquartile range] or n (%)

### Scoring

The higher the score is, the better patient satisfaction, and vice versa. For each individual, the score of each dimension was obtained by computing the mean of the item scores of the dimension. All dimension scores were linearly transformed to a 0–100 scale, with 100 indicating the best possible level of satisfaction and 0 indicating the worst. The global satisfaction score was computed as the mean of the dimension scores.

### Validation of the EVAN-GC scale

#### Validity

Construct validity was measured using principal component analyses with the Varimax rotation test. The translated Chinese version of EVAN-G scale (Table 2) contained 26 items, among which six common factors were extracted through exploratory factor analysis. It includes six dimensions: attention (5 items), privacy (4 items), information (5 items), pain (5 items), discomfort (5 items), and waiting (2 items). As shown in Table 3, the exploring factors analysis of the 26 items found factor loadings of 0.54 to 0.89, with six components explaining 57.8% of the total variance. As expected, correlations between items and the corresponding dimension (item internal consistency) ranged from 0.55 to 0.90, whereas correlations between items and the other dimensions (item discriminant validity) ranged from 0.13 to 0.58 (Table 4).

**Table 2:** The Chinese version of the EVAN-G

No.	Item explanation
	During the preoperative interview with the anesthetist:
1	I got information about anesthesia
2	I was able to ask questions freely about anesthesia
3	I was reassured, relaxed, and confident on anesthesia
	During the preoperative interview with the surgeon:
4	I got information about surgery
5	I was reassured, relaxed, and confident on surgery
6	The surgeon was attentive
	From the first visit until arrived at the operating room:
7	My personal privacy was respected and protected
	When entering the operating room:
8	I felt uncomfortable such as cold, heat, and postured on the operating table
9	My personal privacy was respected and protected
10	The medical staff were attentive
	In the postanesthesia care unit
11	I had unpleasant feelings such as dizziness, nausea, vomiting, and headache
12	I felt uncomfortable such as cold, heat, and postured on the bed
13	I experienced pain
14	I felt pleasant recovery from anesthesia
15	The medical staff were attentive
	Since I back to the ward:
16	I had unpleasant feelings such as dizziness, nausea, vomiting, and headache
17	I felt uncomfortable such as cold, heat, and postured on the bed
18	I had difficulty in daily activity
19	I experienced pain
20	Pain was relieved
21	The medical staff were attentive
22	The nursing staff were attentive
23	My personal privacy was respected and protected
	Summary, since the first visit until today
24	I could see my family and friends often
25	The waiting time of preoperative interview seemed too long
26	The preoperative interview seemed too long

Each item was answered using a five-point Likert scale, where 1 = much less than expected, 2 = less than expected, 3 = as expected, 4 = more than expected, and 5 = much more than expected

**Table 3:** Principal component analysis (Varimax Rotation) of the EVAN-G questionnaire

Item	Item score	Dimension					
		Attention	Information	Privacy	Pain	Discomfort	Waiting
21	3 [3–4]	0.73					
22	4 [3–4]	0.70					
6	4 [3–4]	0.65					
10	4 [3–4]	0.65					
15	4 [3–4]	0.60					
12	3 [3–4]		0.76				
11	3 [3–4]		0.68				
16	3 [3–4]		0.65				
17	3 [2–3]		0.60				
8	3 [3–4]	0.46	0.54				
7	3 [3–4]			0.70			
9	3 [3–4]			0.68			
23	4 [3–4]			0.64			
24	3 [3–4]			0.63			
4	3 [3–4]				0.79		
3	4 [3–4]				0.75		
5	3 [3–4]				0.60		
1	4 [3–4]				0.57		
2	3 [3–4]				0.54		
19	4 [3–4]					0.80	
18	3 [3–4]					0.77	
14	3 [3–4]					0.59	
13	3 [3–4]					0.59	
20	3 [3–4]	0.40				0.54	
26	4 [3–5]						0.89
25	4 [3–4]						0.87

Data are presented as median [interquartile range] or n (%)

**Table 4:** Item-internal consistency, item-discriminant validity, Cronbach’s alpha, and intraclass correlation coefficient of the EVAN-G scale dimension scores and global index

Dimension	Score	IIC	IDV	Cronbach’s alpha	ICC^a^
Attention	72 [64–80]	0.70–0.82	0.14–0.45	0.83	0.75
Information	64 [56–72]	0.58–0.70	0.14–0.33	0.71	0.75
Privacy	56 [48–60]	0.70–0.78	0.22–0.58	0.75	0.85
Pain	72 [56–76]	0.59–0.70	0.16–0.33	0.70	0.63
Discomfort	68 [60–72]	0.55–0.75	0.14–0.33	0.74	0.71
Waiting	32 [24–36]	0.89–0.90	0.13–0.15	0.80	0.68
Global index	69 [65–74]	NA	NA	0.85	0.82

Data are presented as median [interquartile range] and were analyzed by Spearman correlation coefficient. ^a^On a subsample of 36 patients

*Abbreviations***:** *NA* not applicable, *IIC* Item internal consistency, *IDV* Item-discriminant validity, *ICC* Intraclass correlation coefficient

**Fig. 2:**
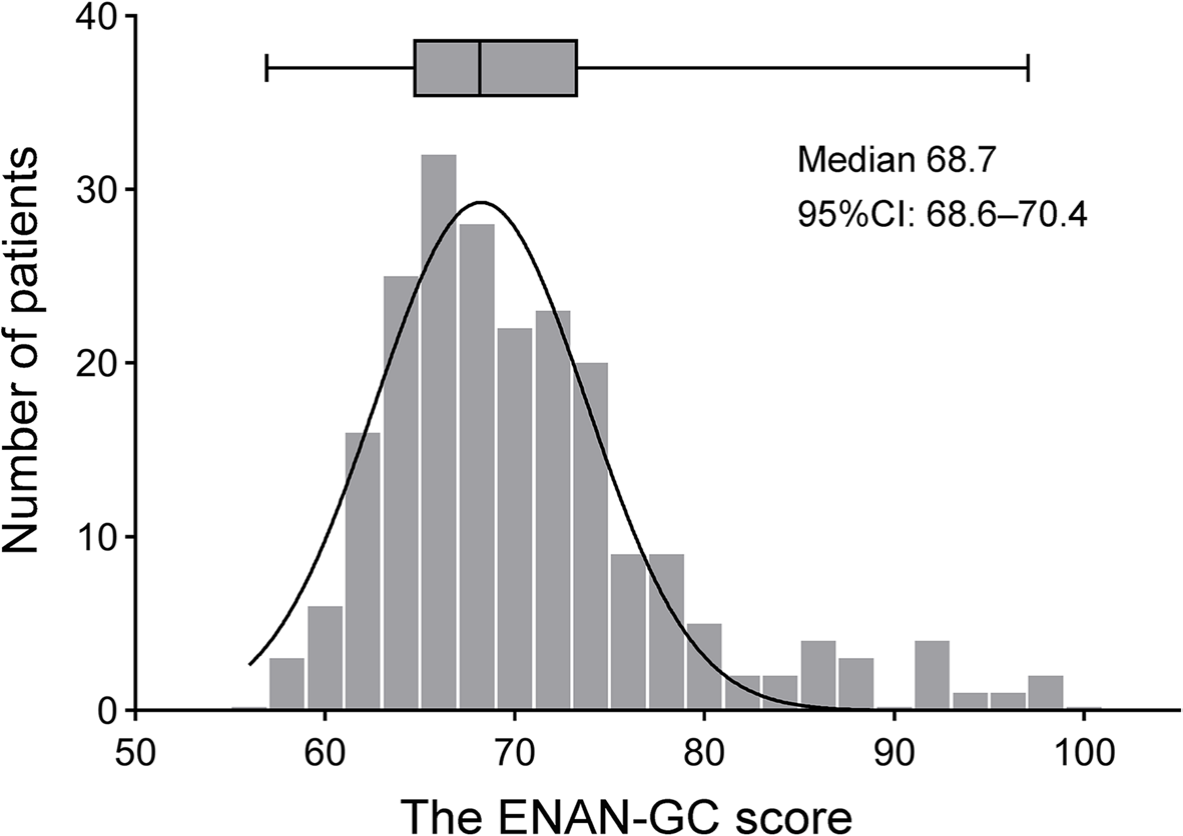
Distribution of Chinese version of EVAN-G scores

The distribution of the 217 postoperative EVAN-GC scores is illustrated in Fig. 2. The details of each item of the EVAN-GC scale are presented in Table 3. The global median satisfaction score was 69 [65–74], and the worst median dimension score was found in the waiting dimension by 32 [24–36] points, while the best was found in the attention dimension by 72 [64–80] points (Table 4). In addition, correlations between the EVAN-GC scale and other concurrent measures confirmed a convincing convergent validity. The correlation between the S-STAI and the EVAN-GC scale global score (*ρ* = − 0.32, *P* < 0.001*)* and the correlation between the MGPQ and the EVAN-GC scale global score (*ρ* = − 0.29, *P* < 0.001*)* were all negative and significant.

As shown in Table 5, except for the EVAN-GC scale poorly differentiated age (*P* = 0.220), findings regarding the differences between the EVAN-GC score and sex (*P* = 0.002), extent of surgery (*P* = 0.045), educational level (*P* < 0.001), patient status (*P* = 0.023), and previous anesthesia (*P* = 0.001) were significant.

**Table 5:** Comparisons of EVAN-G scores according to different statuses

Item	*P* value
Age	0.220^a^
Sex	0.002^a^
Extent of surgery	0.045^b^
Patient status	0.023^a^
Educational background	< 0.001^b^
Previous anesthesia	0.001^a^

^a^Mann–Whitney U test

^b^Kruskal–Wallis test

#### Reliability

The internal consistency reliability of the EVAN-GC scale was satisfactory: Cronbach’s alpha was 0.85. In addition, the test-retest reliability calculated by the intraclass correlation coefficient on a 36-patient subsample was 0.82 (*P* < 0.001).

#### Acceptability and feasibility

Overall, 217 of 220 (98.6%) patients completed the questionnaire in this study. The average time of concluding the EVAN-GC questionnaire was 3.7 [2.9–4.9] min, fully compatible with clinical practice.

## Discussion

A high-quality questionnaire is required for a proper assessment of satisfaction. As the Chinese-language version of the EVAN-G scale based on WeChat has been proven to preserve the solid psychometric properties of the original questionnaire, it has consequently been validated in this study.

This study found that the EVAN-GC scale has similar validity to the French and Spanish versions [8, 13]. Construct validity was acknowledged by correlating the postoperative EVAN-GC scale with each patient’s global rating of postoperative satisfaction.

Regarding convergent validity, we observed a Spearman correlation coefficient between the EVAN-GC scale and the MGPQ (− 0.29) and that with the S-STAI (− 0.32), both of which appeared to be weakly negatively correlated. It has been reported that up to 80.0% of preoperative anxiety could be associated with preoperative information acquisition [23]. Notably, a patient’s state of anxiety (assessed using S-STAI) might be influenced by multiple aspects, such as attention, privacy, and information, correlated with overall satisfaction. The original EVAN-G scale did not appear to be associated with the STAI in any of its dimensions (except for attention), and there was a moderate negative correlation between the STAI and the Spanish version (r = − 0.46, *P* < 0.01), which cultural differences may influence. In short, a correlation was proven between information, attention, privacy, and the global score in this study.

In addition, the differences between the global score and sex (*P* = 0.002), educational level (*P* < 0.001), patient status (*P* = 0.023), the extent of surgery (*P* = 0.045), and previous anesthesia (*P* = 0.001) were significant. In some studies, age was often recognized as a predictor of satisfaction [24]. Some studies of overall satisfaction with medical care show that elderly individuals are more satisfied. However, different studies defined older patients at other boundaries [25, 26]. In this study, we differentiated elderly patients from nonelderly patients at 50 years old, and the results showed that age was weakly related to satisfaction. People with low education levels were likely to have poor cognition of health. It was reported that people with lower educational experiences had lower cognitive levels of health and lower satisfaction levels than others [23]. The EVAN-GC scale confirmed that differences between many groups were meaningful, reflecting a relevant discriminate validity. Overall, the recently translated scale had acceptable discriminant validity.

Moreover, the reliability of the EVAN-GC scale was similar to that of the initial validation and was classified as excellent. The Cronbach’s alpha was significant (0.70–0.85), where a score of 0.7 or higher is acceptable [27]. In addition, the translated questionnaire’s test-retest reliability (0.63–0.85) was satisfying and stable, exceeding published recommendations (correlation 0.60) [28]. Compared with missing data of the original EVAN-G scale in each dimension (0.6–2.7%) and 1.7% of that in the Spanish version [8, 13], this study reported no missing data after detailed instruction, which was due to the convenience and portability of social application—confronted with a lower average duration of completing the EVAN-GC scale (approximately 4 min) than those of the initial questionnaire (approximately 9 min) and Spanish version (approximately 14 min), which showed excellent acceptability.

Admittedly, the experiment has several limitations. First, the study was carried out in one of the most tertiary hospitals in China. Therefore, the generalization of the conclusions to other contexts requires further validation in smaller-scale hospitals, different regions, and other cultures, even if they are in the same languages. Because cultural differences might unconsciously influence the applicability of the questionnaire, it must be adapted to the context in which it will be administered. Second, already present in the original version of the EVAN-G scale, it only refers to procedures under general anesthesia and is related to certain surgical specialties. It was reported that evaluating patient satisfaction with a specific intervention or in-study participation is crucial for the research community [29]. Other questionnaires should be applied to measure a satisfactory condition when regarded with interventions under locoregional anesthesia or monitored anesthetic care. More extensive validation of the questionnaire is advisable to endorse applicability to surgical specialties other than those mentioned in the study.

## Conclusions

Our results reveal that the EVAN-GC scale preserves the psychometric properties of the original French questionnaire. The EVAN-GC scale is a valid and reliable tool for assessing patient satisfaction in the Chinese population.

## Data Availability

The datasets used and/or analyzed during the current study are available from the corresponding author on reasonable request.

## Abbreviations

EVAN-GC: The Chinese version of the EVAN-G
S-STAI: The Short version of the Spielberger State-Trait Anxiety Inventory
MGPQ: The McGill pain questionnaire
LPPS: Leiden Perioperative Care Patient Satisfaction
IQOLA: International Quality of Life Assessment
ISAS: The Iowa Satisfaction with Anesthesia Scale
NA: Not applicable
IIC: Item-internal consistency
IDV: Item-discriminant validity
ICC: Intraclass correlation coefficient
